# Why less may be more: a mixed methods study of the work and relatedness of ‘weak ties’ in supporting long-term condition self-management

**DOI:** 10.1186/1748-5908-9-19

**Published:** 2014-02-13

**Authors:** Anne Rogers, Helen Brooks, Ivaylo Vassilev, Anne Kennedy, Christian Blickem, David Reeves

**Affiliations:** 1 NIHR CLAHRC Wessex, Faculty of Health Sciences, University of Southampton Building 67, Highfield Campus, University Road, S017 1BJ Hampshire, UK; 2 NIHR CLARHC Greater Manchester Institute of Population Health, University of Manchester, Williamson Building, M139PL Manchester, UK

**Keywords:** Self-management, Weak ties, Social networks, Long term conditions, Survey, Qualitative, Chronic illness work

## Abstract

**Background:**

The distribution of the roles and responsibilities of long-term condition management (LTCM) outside of formal health services implicates a wide set of relationships and activities of involvement. Yet, compared to studies of professional implementation, patient systems of implementation remain under-investigated. The aim of this paper is to explore the work, meaning and function attributed to ‘weaker’ ties relative to other more bonding relationships in order to identify the place of these within a context of systems of support for long-term conditions.

**Methods:**

This is a mixed methods survey with nested qualitative study. A total of 300 people from deprived areas in the North West of England with chronic illnesses took part in a survey conducted in 2010 to 2011. A concentric circles diagram was used as a research tool with which participants identified 2,544 network members who contributed to illness management. Notions of ‘work’ were used to describe activities associated with chronic illness and to identify how weaker ties are included and perceived to be involved through social network members (SNM) contributions.

**Results:**

The results provide an articulation of how SNMs are substantially involved in weak tie illness management. Weaker ties constituted 16.1% of network membership involved in illness work. The amount of work undertaken was similar but less than that of stronger ties. Weaker ties appeared more durable and less liable to loss over time than stronger ties. The qualitative accounts suggested that weak ties enabled the moral positioning of the self-managing ‘self’ and acted on the basis of a strong sense of reciprocity.

**Conclusions:**

Weak ties act as an acceptable bridge between a sense of personal agency and control and the need for external support because it is possible to construct a sense of moral acceptability through reciprocal exchange. Access to weak tie resources needs to be taken into account when considering the ways in which systems of health implementation for chronic illness are designed and delivered.

## Introduction

Professional systems of implementation including those for long-term conditions have drawn on the idea of social networks to make sense of how care is mobilised and delivered to patients [1]. This interest extends to ‘weak’ tie networks, which are viewed as an engine for the coordination of key activities, collaborations and interactions that create and facilitate pluralist, informed and positive support [2]. The growth of patient and public involvement and the rise in the importance attributed to self-management for long-term conditions^a^ supports a shift in analytical focus to patient systems of implementation, which have the potential to act as a complement or alternative to traditional health service provision. The rationale for such a focus is reinforced with recognition that patients with long-term conditions spend relatively little time in contact with health professionals in comparison to the activities needed to manage long-term conditions in everyday life [3]. Mapping the configuration of patients’ personal communities of support is relevant for understanding who does what, how and why for long-term condition management. It also has implications for policy makers in planning investments in professional delivery of chronic illness management support versus looking to invest in support coming from a much broader set of illness management relationships.

The equivocal evidence of widely advocated programmes of self-management support (SMS) based on enhancing individual capacities to acquire skills and knowledge to enhance chronic illness management [4,5] has led to suggestions that increasing effective targeting and implementation of SMS requires a focus on connections to resources that might enable access and mobilisation of resources and opportunities for SM in domestic and community settings [3,4,6]. Social network analysis (SNA), which is concerned with the structural arrangements and content of social relations and positions (a set of actors linked into networks), has potential for viewing the chronic illness management (CIM) work force as a system of networked support for personal management [7-9].

The distribution of the roles and responsibilities of long-term condition management (LTCM) between groups of involved actors outside the immediacy of the health service implicate a wide set of relationships and activities of involvement. There is increasing knowledge about how lay expertise can act as an embedded resource for some but make it difficult for those with limited resources to mobilise appropriate support [10-12]. Salience has also been attributed to the strong tie relationships of intimate others, mainly in exploring the dyadic relationships of partners, or parents and children, in providing care. Most of what is termed ‘informal care’ in the literature focuses almost exclusively on how much support close relatives provide in the way of care and emphasising the relationship with ‘formal care’. Established commitment and connectedness to sustain caring long-term relationships with close family members are based on strongly held normative values that the other will meet unfulfilled needs, feelings of intimacy, cohesiveness, and a sense of filial belonging and obligation [13]. Important as these relationships are, one of the consequences of the focus on dyadic relationships of intimate lay caring relationships is that the wider contributions that may be being made by a broader set of actors, resources and technologies remains under-acknowledged [14].

### Mapping weak tie involvement in chronic illness relationships

The case for exploring ‘weaker’ ties in the context of SMS is supported by the recognition that a salient feature of contemporary society is a less centralised and broader diffusion of support networks and distributed knowledge that has grown alongside or outside of a ‘primary’ set of intimate relationships [6,15-18]). A growing interest in the role and function of weak ties has been linked to a recognition of the fragmentation of social life, generating a complex set of impersonal, transient second level networks and associations made up of neighbours, work colleagues, and taxi-drivers. In contrast to the strong bonding ties of intimate others, weak ties constitute a small proportion of all exchange relationships (10%) [19]. Typically, weak ties are characterised by the briefness of interactions with acquaintances and strangers based on lower levels of trust, commitment and connectedness than more bonding stronger ties. Nonetheless, in a number of areas, they represent important sources of support and are attributed with the power to enhance the reach and cohesion of social relations. They act, for example, as an effective conduit for accessing valuable job opportunities [20-22] and for the urban poor in ‘making it from one day to the next’ [23]. The experience of chronic illness literature implicitly suggests that less intimate, more distanced contacts (‘network of networks’) may offer preferential or different facets of support [24]. For example, online relatedness can provide for a more distanced, less stressful engagement than offline intimate and proximate relationships [25], and the search for intense support in the early stages of a condition has also been shown to give way to the mobilisation of a wider network as the illness progresses [26].

Here we extend a previous focus on the ‘strength’ of weak ties to look at how they are construed and what function they perform in relation to the work of long-term conditions management within personal systems of support (consisting of the person with the condition, members of their personal network, community groups, health professionals, and non-health professionals). Weak ties are sometimes characterised as limited to providing specific types of help, consisting of individuals who are not interpersonally close, but with whom people interact in a limited way, inferring restriction to non-kin relationships. However, Bott has indicated that not all family member relationships are strong primary sources of support and that some are more accurately described as weak ties [27]. This may also be the case for the input of some professional health workers. Therefore, definitions relevant to exploring weak tie relationships should avoid being constrained by social positions or roles. Here we ascribe in the main to Bott’s definition (whilst noting a tension in who or what constitutes a ‘weak tie’ evident in participants’ accounts identified in the empirical data presented below).

The types of support identified in this study are relevant to and constitutive of different ties and include instrumental, emotional, and illness-related work/support [28]. We adopted an approach in which personal communities are used to examine how a person at the centre of a network implicates the members of her/his egocentric world who are involved with long-term condition management. Here we aim to clarify the attributed meaning and nature of weak tie relationships experienced by respondents with reference to identity construction and other sources of SNM (social network member) support to which people have access. We look firstly at how people ranked weak ties relative to others, drawing on empirical data to explore their role and function as elements of chronic illness ‘work’ within whole networks of people with CIM [4,29]. We then move on to consider qualitative data related to narrative constructions of weak tie involvement.

In relation to the former, we used three different domains of chronic illness work:

1. ‘Illness (specific) work’ refers to the work related to: taking medications; regimens of monitoring; understanding and responding to symptoms; and making appointments.

2. ‘Everyday work’ refers to: the tasks of housekeeping and repairing; domestic and occupational labour; child rearing; support and activities related to diet and exercise, general shopping and personal care.

3. ‘Emotional work’ refers to the work related to comforting when worried or anxious about everyday matters, including health, well-being and companionship. It also includes a biographical dimension associated with the reassessment of personal expectations, perceived capabilities and future plans, personal identity, relationships and biographical events.

## Methods

### Design and study participants

A cross-sectional mixed methods study was conducted between April 2010 and January 2011 incorporating a postal questionnaire and a face-to-face network interview (the full description of study design is published elsewhere [29]). A total of 2,001 patients with chronic heart disease (CHD) or diabetes were randomly selected from the disease registers of consenting GP practices in deprived areas of NW England and were sent invitation letters. We chose deprived areas on the basis that deprived populations have most to gain from self-care support resources and thus it was important to identify existing support for those who might benefit the most from any prospective intervention. A total of 300 people responded to invitation letters and completed both elements of the study^b^. Data on network members was captured and mapped using the method of concentric circles of importance. Participants were requested to map social network members using a diagram consisting of three concentric circles [30]. Responding to the question, ‘Who do you think is most important to you in relation to managing your condition?’, network members placed in the central circle were those considered most important, members placed in the middle circle were considered less important than those in the central circle, and members in the outer circle were considered less important than those in the two inner circles. Participants were permitted to place as many network members as they wished, of any type of relationship they considered relevant (*e.g*., family, friends, medical professionals, pets), including groups and services (*e.g*., workplace, religious group, food delivery service), as well as individuals. The face-to-face interviews provided an opportunity for initially overlooked network members to become visible during the discussion, and for detailed information to be collected about key attributes of each network member and the contributions they make to different sets of illness-relevant tasks. The study was designed to identify how weaker ties are included and perceived to be involved in SNM contributions to illness work. Weak tie involvement was identified through identifying and describing the members who make up the social networks of personal communities of individuals and how they were valued in importance, combined with the illness ‘work’ undertaken. This was understood as the contribution of network members to various activities (encompassed under three domains: illness-specific, practical and emotional work^a^).

For the purpose of the analysis, we designated those whom participants placed in the outer of the three concentric circles as being ‘weak ties’. Follow-up took place 12 months after baseline data collection (this achieved a response rate of 76% [n = 248]). Data collection in the follow-up stage was via a postal questionnaire. To collect social network data at follow-up, a self-report grid was used that listed, for each participant, all the network members they identified at baseline, for each of which the participant (i) indicated whether the member was still part of their network and (ii) rated the work currently done in each domain on a 1 to 5 scale. Participants were asked to also list and rate any new members of their network.

### Quantitative measures

We produced questions relating to each category of work to capture the role of different network members from the perspective of the individual. For the purposes of the analysis, categories were combined. During the interviews, participants were asked to elaborate on the roles of network members by rating each between 1 and 5 on a Likert scale for 17 different aspects of work undertaken by members, where 1 is ‘not at all’ and 5 is ‘a lot’.

In addition, data was also collected that measured the types of relationship present in each person’s network: the perceived closeness of network members, the size of the network, and the fragmentation and density associated with individual networks. Results showing the articulation of how social network members overall are substantially involved in illness management and further details about the method and tools used are presented elsewhere [29].

### Quantitative analysis

Member scores on types of work were analysed using a multilevel linear regression model, with members clustered within networks and network means treated as a random effect.

Relationship ‘type’ was included as a set of ‘dummy’ explanatory variables. To compare weak ties directly against other types of ties within the same network, we restricted the sample for analysis to only those networks that include a weak tie (n = 177)^c^. The 177 networks with at least one weak tie contained 1,698 members in total.

### Qualitative interviews

A semi-structured interview formed part of the survey to further explore the roles of individual network members, and the interview questions can be found in Table 1.

**Table 1:** Breakdown of weak ties

		**Frequency**	**Percent**	**Valid percent**	**Cumulative percent**
Valid	Partner or spouse	2	.5	.5	csl
	Close family	51	12.4	12.4	12.9
	Other family	15	3.7	3.7	16.6
	Friends, colleagues or groups of friends/colleagues	116	28.3	28.3	44.9
	Pets	16	3.9	3.9	48.8
	Medical professionals	112	27.3	27.3	76.1
	Groups	75	18.3	18.3	94.4
	Other	23	5.6	5.6	100.0
	Total	410	100.0	100.0	

The broad focus of the interview was on participants’ management of long-term health conditions (diabetes and chronic heart disease) and how social networks and relationships were described.

### Qualitative analysis

The qualitative interviews allowed further elaboration of the meaning and contribution of relationships to networks and the nature of the context and content of illness work undertaken. Participants elaborated on their answers to the pre-determined questions, forming the basis of the quantitative data. For the analysis here, we purposively selected those who identified outer circle member contributions to illness work. The interviews were audiotaped with participants’ consent, transcribed verbatim and analyses assisted by Atlas (version 6). A framework analysis was undertaken, coding data relating to the work (emotional, illness, or practical) implicating weak ties narratives (outer circle). The researchers coded transcripts independently and then met to discuss, examine, and agree on emergent codes. A list of final themes and related sub-themes was produced. We identified themes related to the nature of weak ties which were considered in the context of narratives about illness work-related relationships, relatedness and peoples’ sense of self.

### Quantitative results

#### ***Constellation and distribution of outer (weak ties) versus inner (stronger ties) network members***

**Figure 1:**
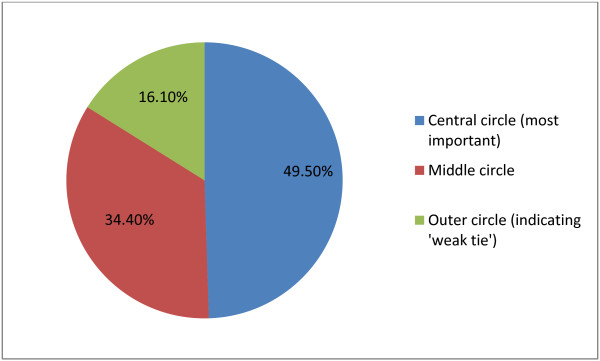
Distribution of ties within the social network reported by participants.

The 300 participants included in the study identified 2,544 network members who were seen to be important to them in terms of long-term condition management as defined by placement in one of the 3 concentric circles [30]. A total of 1,259 (49.5%) network members were placed in the central circle of the diagram (those considered most important), 875 (34.4%) were placed in the middle circle (those considered less important than those in the central circle), and 410 (16.1%) were placed in the outer circle (considered less important than the two inner circles, Figure 1). These 410 network members constitute an indicator of weak ties (along the lines of the suggestion by Bott; see above). Of these 300 study participants, 177 (59.0%) reported having at least one weak tie in their network. The number of weak ties within networks ranged from one to seven. Thus weak tie involvement is a much smaller proportion of the total number of relationships providing support. The people identified for placement in the outer circle indicated weak tie involvement (See Table 2). For the largest part, this constituted friends, colleagues (28.3%, n = 116) and health professionals (27.3%, n = 112). To a lesser extent, they included voluntary groups (18.3%, n = 75), pets (3.9%, n = 16) and non-close kin relationships (3.7%, n = 15). A total of 12.4% were close family members. Only two spouses were located in the outer circle.

**Table 2:** Breakdown of weak ties

**Personal community member**	**Frequency**	**Percent**
Partner or spouse	2	.5
Close family	51	12.4
Other family	15	3.7
Friends, colleagues or groups of friends/colleagues	116	28.3
Pets	16	3.9
Medical professionals	112	27.3
Groups	75	18.3
Others	23	5.6

**Figure 2:**
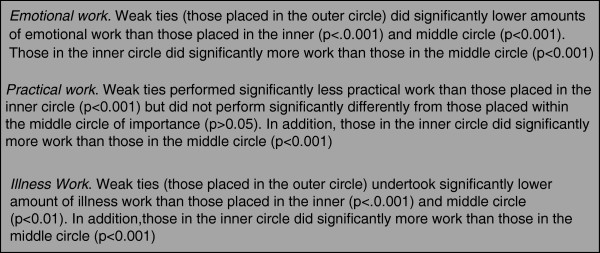
Results from multivariate linear regression.

Table 3 summarises the mean amount of different types of work undertaken by different types of ties within the networks^d^. A number of statistically significant differences were found between weak ties and other types of ties, and these are summarised in Figure 2. Weak ties undertake significantly less emotional and illness work than those in the inner and middle circles, but only slightly less practical work than those in the middle circle.

**Table 3:** Mean emotional, practical, biographical work scores for different relationship categories

	**N**	**Group mean (standard error)**	**p-value**
***Emotional work***			p < 0.001
Outer circle	410	*2.14 (0.15)*	
Middle circle	572	*2.99 (0.12)*	
Inner circle	715	*4.70 (0.11)*	
**Total**	1698		
***Practical work***			p < 0.001
Outer circle	410	*0.53 (0.11)*	
Middle circle	572	*0.78 (0.09)*	
Inner circle	715	*2.24 (0.08)*	
**Total**	1698		
***Illness work***			p < 0.001
Outer circle	410	*0.98 (0.11)*	
Middle circle	572	*1.43 (0.10)*	
Inner circle	715	*3.11 (0.09)*	
**Total**	1698		

### The dynamics and durability of weak tie ‘work’

Social network dynamics refer to the degree to which specific SNMs move in or out of a personal network [31]. Trajectories of chronic illness are inevitably accompanied by changes to personal communities due to changes over time in the experience of a condition and available means of support. The relative stability of weak compared to strong ties with less of a risk of erosion is implied from some results of the quantitative analysis^c^. Weak ties are more durable as judged by a slower rate of loss than stronger ties (*e.g*., only 27 [1.1%] network members were lost from the outer circle [indicating a weak tie] as compared with 60 [n = 2.4%] from the middle circle and 37 [1.5%] from the central circle). The key properties of weak ties from the data are that they are involved in less work than intimates, and the subjective assessment of individuals places them below the middle ranking members (even if objectively they do similar rates of work). Weak ties appeared more durable over time (for a year) and more so than for inner ‘strong ties’ circle members^d^.

### Qualitative results

The qualitative data illuminates more regarding the meaning for individuals of weak ties in the context of relational work and identity work associated with the ‘self-managing self’. Participants in our study described instances of less intense and intimate interactions that occur in and outside of their regular routines and beyond the home, suggesting that weak tie exchanges in the context of chronic illness take place in a broad environment.

### Attributing the work and status of weak ties

Respondents provided an overall indicator of the level of involvement and attributions of importance through placement across three circles. Here, our focus is on the outer circle contributions and relationships. However, it is relevant to note that weak tie relationships were more frequently mentioned in interviews than were actively able to be included in the mapping exercise and, therefore, the quantitative data are likely to be an underestimate of their involvement. The reasons for this relate to the downplaying by respondents of their relevance for chronic illness (*e.g*., they are cast as friends for leisure purposes) or classified as potential support to be accessed in the future (*e.g*., friends at the local shop and/or a neighbour who is felt would be there in an emergency and who has a spare key to the house [ID141, male, 73, Diabetes, CHD, and CKD]). In terms of who was placed where in the circle schema, it was clear that those who were more usually found in the inner circles as bonding strong ties (*e.g*., professionals or close relatives) could end up in the outer circle due to a dilution of or dissatisfaction from these relationships. For example, for one respondent, regular contact over a lengthy period of time with a clinic for a chronic condition is the only entry made to the outer circle, and placement relates to requests for help with information that never emerges:

If we see anything new, like if I’m on the Internet, looking at things… (I mean grandchildren), they’re on computer, so if they hear anything new, they tell me and we go [to the clinic] and see if it’s any use to me, you know. Then we usually tell (the diabetic nurse), don’t we, and she says she’ll look into it, but…. it never materialises, does it? She keeps saying I’ve got it well under control but I don’t feel as if I have, I really don’t feel as if I have. I never found out what were the matter with me, did I (from them)? (ID188, female, 80, Diabetes and CHD).

Sometimes it was the level of work that could not be provided by close relatives that provided the basis of outer circle placement. This could be due to less proximate availability, meaning more infrequently undertaken work, or the inability of undertaking just in time help – a precondition of the more intense caring roles characteristic of bonding tie members of the network.

Yes I’ve got more grand children…. You know, they all… well they’re all important to me, my grand children…. But they don’t, they’re not here all the time, like my grand daughter, she comes every night on her way home from work… you know to make sure I’m OK… (ID055, female, 79, CHD and COPD).

The size of the overall network including weak ties from within a personal network mattered. Weaker ties in networks with multiple members including those that provided a lot of support in the first two circles seemed to justify weak tie attribution on the bases of comparisons of less importance and/or less input relative to others. In some instances, weaker ties were clearly secondary and did not operate outside a connection with more numerous bonding ties – for example, in relation to the co-option of a significant other in obtaining personal or domestic care. Additionally, the nature of such work could be relatively fleeting, less intense or intimate, as in doing the garden or cleaning once a week or in the provision of time-limited instrumental support (*e.g*., the provision of goods and services, such as carrying a bag of shopping; helping with mobility; etc.).

Weak tie involvement can provide a means of managing relationships and work of more intimate ties. In relying on outer circle input, fewer demands may be placed on closer network members providing a lot of support. Weak ties are useful when help from a stronger tie would be inconvenient, impractical or unwanted. However, in a number of instances, it is also the case that quite disabled people managed well with mainly weak ties. One such person who had difficulty seeing to cook had daily routinised contact with a range of food outlets, which he compares favourably with the unreliability of official caring agencies and the benevolence of strong ties (in this case a sibling).

That’s how I go on, like today, I go down into H, I usually catch the five to seven bus in the morning and I go into the Mall and there’s three young ladies there. She cooks me my breakfast, my breakfast consists of a bacon muffin with raw onion on and a couple of cheese slices, which I put on myself….. Every day I go down there seven days a week. I don’t get my breakfast there on a Sunday, I go into Wetherspoons on a Sunday and get a breakfast there. Oh, I’ve got friends all the way around there that I can talk to and everything (ID094, male, 78, Diabetes and CHD).

The work, relationships and activities of the outer circle members can fulfil mental health needs in terms of a bridging function. This example of a friend enables this person to access other activities away from family and health professionals.

R: I don’t go anywhere. I go fishing twice a week. N takes me… it’s a friend I’ve pointed out, he lives about four or five doors away.

I: How much… would you say going out, kind of, getting fresh air, helps you manage your diabetes a bit?

R: Well I don’t think it manages it, but I’ve got to manage it myself. It does help, you know psychiatric type of thing… (ID298, male, 80, CHD).

### Enabling the moral positioning of the self-managing self

The tendency to attribute less importance to the outer circle members in narratives reflected the more fleeting contact and input one expects from weak ties. A taken-for-granted attitude was evident in the way in which the respondent conceived of the primacy of their own role. Voluntary or community groups invariably appeared in the outer circle and permitted the adoption of a ‘take it or leave it’ attitude on the part of the respondent in acknowledging their significance relative to specific individuals. This is illustrated in this account of church attendance (placed in the outer ring). The church had identified functions, but its importance was marginalised both in the way it was spoken about and in terms of minimising, with reference to not attending more often and in providing help in return.

R: I’m a bit isolated here on my own, you know? But I don’t really see a lot of people. I mean I belong to a church but I don’t get any practical help. I just to go to church, that’s all.

I: Okay. Well… but that’s quite important to you to stay in touch with the church then?

R: Oh yes.

I: Okay. Well that’s a form of support. I think that’s…

R: Yes. Yes, it is and the Minister comes around occasionally. And if I needed help I think I could get it from there. I just go Sundays as a rule and then try to live alright, you know, in between times. But no, I’m not… it isn’t that I’d like to go more…. And just now, (the minister) is trying to make money for repairs, so I’m helping where I can there, you know. But no, I don’t wish I could go more (ID181, female, 92, Diabetes and CHD).

Weaker ties were frequently talked about as an afterthought – as ‘obviously’ of not much importance. Minimising the importance of the help received from weak ties in discussion with the interviewer seemed to be a means of preserving the socially normative requirement of ensuring that the input of close relatives was not overlooked. Here, an explanation of the importance of a discrete and needed task (driving) is embedded in an account of the awareness of the status of not being kin:

Well that’s my close, these are my close, what I would call close friends. These are very important, because I don’t drive. … They’re friends, they’re not relatives.

Now *these* two have been my backbone for thirty, forty years, they are my next of kin (ID154, female, 76 years, Diabetes and CHD).

Self-efficacy, stoicism and not wishing to be seen as dependent on others are markers of the experience of illness, and to be self-sufficient is one way of protecting what Bury has termed ‘meaning at risk’ [31]. Establishing legitimate self-hood as a good self-manager [10] involves emphasising control over personal decisions, being stoical in the face of adversity, and giving primacy to a sense of self-worth, autonomy and independence. This moral positioning, together with the relational work of negotiating the acceptance of assistance^e^, is reflected in the input of weak ties, which act as mediators between the imperative for projecting a sense of control and mastery and the need for support to assist with tasks. In other words, weak ties encompass a tension between the need to be seen as managing adequately without the help of others, as at the same time needing to accept support from others. Keeping people who want to help too much at arms-length is one strategy for doing this, and weak tie involvement is one way of achieving this. Intimate relationships with close ties can threaten to overpower through a combination of the debilitating effects of long-term illness coupled with overstepping established boundaries of maintaining a level of independence:

I: Yeah. And do you think they’re both. . . they’re all very important in terms of supporting. . .

R: Oh they’re very good. Oh yeah. Definitely. Definitely. I went away and when I come back they’d redecorated for me. I’ve only got to pick the phone up and say I want something, or if I go out and I’m at the shops they phone, ‘well why are you at the shops, why haven’t you told us, we’ll take’. . . I said, ‘no, I’ve got to have a bit of independence’. Sometimes they overpower you but I can’t tell them that because they’re so good. Yeah. I try to sneak out now and again. They don’t like me doing it, but I’ve got to have a bit of independence (ID248, female, 71 years, CHD).

Placement in the outer circle meant that network members were attributed with distance, allowing the individual to project a sense of self-reliance. Alternatively, everyone who helped was put in the outer circle because respondents (mostly men) thought they were managing their health on their own without any input from anyone, even when it was apparent during the interview that this was not the case. Thus the notion of ‘managing by myself or my family’ was one way in which the roles of weak ties were partly hidden. In contrast, the placing of family members in the inner circles signified bonding ties of great importance: ‘well, friends come and go, but family is always there’.

The portrayal of weak ties as being of limited importance fitted with the projection of an image of not requiring too much in the way of assistance, balancing a loss of capacity with the retention of a sense of personal control. The simplicity and fleeting nature of the tasks characteristic of weak ties enable a person to keep ‘life on track’ (*e.g*., by a neighbour getting the paper and doing the shopping). The provision provided by a weak tie can also be portrayed as something normally done by the person themselves and thus easily accounted for in the normalcy of talk about managing things as much as possible by oneself.

R: I need a cleaner and I need the gardener you know–

I: And would you say they help you with the management of your illness?

R: Only that they do my work, don’t they, so I would say yes. I’d like to be able to toddle around to the shop and get the paper, or I’d like to be able to decorate my house, as I used to do. But I just can’t! (ID055, female, 79, CHD and COPD).

### Weak ties as the bases of reciprocity and personal exchange

The independence of managing by oneself together with the low level input of work by weak ties are accompanied by accounts of reciprocity. This is clearly articulated by ID233, who framed contact with an outer circle group of trainee healthcare workers by fore-fronting what she was contributing to the relationship:

I go there once a year to have a chat with the physiotherapy students, and I’m really happy to [do] that because it’s easy for you, you know, it doesn’t… it’s not taxing or nervous for me, I don’t mind doing things like that, plus the students seem to get quite a lot out of it, they always feels it’s interesting to speak to someone, who’s got a condition, rather than just read about it. So, I guess, that helps me in turn, because I get some feedback from them, so I’d probably put that in the outer circle as well (ID233, female, 45, Diabetes).

The existence of a cash nexus as a component of reciprocity acted to provide legitimacy that came from providing a service rather than providing ‘help’, as a family or friend would. Some people described meeting need in this way from people they do not classify as knowing particularly well. Cleaning services and taxi drivers featured strongly in this regard as in this account of paid help:

But probably I do admit that I’m very independent, and it takes me a lot to ask for help, but help I’ve had to ask for in the last two years. It does go against the grain but one has to be humble sometimes… At the moment I’m able to more or less work things out like that for myself, because I know I couldn’t clean through the flat all in one go, small though it is, so I split it up. That sort of thing, I’m a person that can plan it to fit in with my health requirements or downfalls (ID154, female, 76 years, Diabetes and CHD).

Trust was also a part of such reciprocal relationships.

I: OK, how did you get to know [a taxi driver whom he has known for four years]?

R: Well he’s a trusty person. He taken me around. He go to post office which I don’t have to go there. If I would sit here and phone the bank and tell them [taxi driver] coming, they would accept him because he generally take me there and they know me, so he’s a trustworthy person (ID309, male, 96, Diabetes and COPD).

I said [to social care worker assessing needs] I’ve managed to get out even in the deep snow because of the taxi companies, I know who will send a cab for me and I pay a taxi fare to go down and I’m out maybe for an hour, I might have a drink in Hyde and then I’m back within an hour. And that’s all I ever got. I got more help from the taxi companies [than social services] in the deep snow, they used to hold my arm and carry me…. Got me across it to make sure I didn’t fall and the same going out to make sure I didn’t fall whilst going out… I get more help from people like that and I’ve got more enjoyment out them that what I have from anybody else (ID094, male, 78, Diabetes and CHD).

The low level of commitment and expectations that are often associated with weak ties means greater tolerance of periods of discontinuity and is one of the reasons that weak ties are more durable than strong ties (see above), as indicated by the intermittent contact with visiting door step visitors (*e.g*., Jehovah’s Witnesses).

R: The Mormons, they have quite some very interesting ideas on religion, which don't quite fit [chuckles].

I: [chuckles] But when he used to come here he would come once a week?

R: It’d be about once a week, yes. But as I say, I’ve been either elsewhere or at the hospital. But he did know I’d been ill, so he’d probably… I expect to see him anyway (ID046, male, 76, Diabetes and COPD).

## Discussion

Network support for long-term conditions has often been framed in a way that emphasises the central role of key care givers such as professionals and close relatives. Support from others outside of these two groups has rarely been explored [32,33]. This study illuminates the hidden potential of more marginal agents to supporting long-term condition management. It shows something of the function and value of the input of weak ties to long-term condition self-management in terms of both structure and meaning. Whilst a lack of familial support can be substituted by others such as professionals or lay others, our data show here how weak ties should be seen as more than this. Analysing weak ties within a larger system of chronic illness management illuminates role differentiation and interaction. Weak tie members provide a broad and qualitatively different nature of input (in comparison to more binding ties or the prescribed and focussed input of formal health systems). How certain individuals within a personal network are placed in the outer circle of support implied a number of different meanings. The latter ranged from placing professionals, more usually thought of as the centre of support, at the margins because of dissatisfaction with their response, to individuals providing support who were considered to be de facto of less status than others (*e.g*., pets, taxi drivers) and those providing less traditionally highly valued work of more frequency and intensity.

At a structural level, we showed that ‘weak ties’ constitute less than 20% of total network members and undertake less of similar categories of work than others within a heterogeneous network. In terms of network dynamics, outer circle network relationships are more durable in terms of being able to be sustained over time.

However, in this context of self-care, they are different from those portrayed in other areas of social life. The relationships are less transient, and may involve more connectiveness (emotional closeness and frequent contact). Whilst in terms of a theoretical basis for both the quantitative and qualitative analyses, we made the decision to designate those placed in the outer circle as ‘weak ties’, the qualitative data highlighted the complexities and tensions of sticking rigidly to this. Some of the friends and acquaintances traditionally deemed as ‘weak ties’ were placed in the inner circles rather than the outer circle and vice versa. Even though this was pitched against evidence of weak tie support keeping peoples’ lives on track, our data showed that weak tie outer circle attributions were made according to perceptions of doing less important work, a weaker sense of intimacy, dependence, intensity and frequency of contact than was the case with stronger ties. In this respect, accounts of weak tie placement were hidden from view, and participants understated the value of weak tie involvement in favour of narratives of self-reliance. Nonetheless, weak tie relationships provided discrete and useful functions – transport, spiritual support (church or place of worship), in a way that was generally trusted. This suggested that weak ties are valued precisely because they did not implicate burden and felt stigma about the receipt of help associated with the intense involvement of closer ties pervading more intimate caring relationships [17]. Thus, weak ties seemingly play a role in the context of long-term illness management that is central to a struggle to maintain a sense of control and agency. Weak tie input was less imbued with idioms of guilt and shame and the need to manage a ‘crisis of credibility’ that more usually accompanies the role of being chronically ill in the face of others. Similarly, reciprocating actions (*e.g*., payment or participation in the church, or through being a friend) are markers of mastery and control. In this respect, they render the dependence/independence balance easier to negotiate when input is less intense, more fleeting and discretely demarcated by time and place. Weak tie relationships seem to avoid the intense ambivalence and negative sides of feeling/being too dependent on intimate others precisely because it is possible to construct more of a sense of reciprocal exchange. This reciprocity and the potential to offer respite from the negative aspects of intimacy may account for why weak tie relationships have been found to be experienced as less stressful [34], and in the context of self-care they may be more durable and sustainable than other ties. This analysis points to the need to distinguish between strong and weak ties and suggests the need for substantive differentiation of functions and relationships in a self-care context. A useful distinction might be one that differentiates between discretionary reciprocal relationships (weak ties) versus indispensable ‘dependant’ (strong ties).

### Limitations

The study sample was drawn from English general practices in deprived areas. This suggests that there are limitations to the typicality of the findings to other settings. For example, in other cultural contexts, the importance and function of weak network ties might differ. Among African-Americans, weak tie connections monitoring health stemming from church membership seems to have greater salience [34-36]. The limitation of using the concentric circle method was that at times, weak tie connections were conflated with stronger ones that people were dissatisfied with. This suggests a need to focus on differentiating more clearly between strong and weak tie contributions and between different types of weak and ‘very’ weak ties in the context of self-management support.

## Conclusion

Weak tie relationships in long-term condition management seem to act as an acceptable bridge and mediator between a sense of agency and control and the need for support. The very fact that accounts dismiss, marginalise or incorporate weak tie involvement as part of self-management illuminates the strength of weak ties in chronic illness management. Implementing self-management support through engaging patients is an increasingly normative expectation of professional practice [23]. This research suggests that professional systems of implementation may benefit from incorporating the importance of weak tie relationships in strategies for management. Formal systems of support may need to incorporate ways of linking patients into networks of support that extend beyond close relatives’ involvement to include broader environments and resources implicating weak tie relationships. Such approaches might seek, as a primary objective, to link into specific sources of support from within the networked relationships that make up patients’ personal systems of support. One such strategy is incorporated into the development of a community referral tool predicated on network mapping that facilitates access to community-based and weak tie resources undertaken as part of the programme of work presented here [37].

## Endnotes

^a^A long term condition is defined by the Department of Health ‘as a condition that cannot, at present be cured; but can be controlled by medication and other therapies. Examples of Long Term Conditions are diabetes, heart disease and chronic obstructive pulmonary disease’. http://webarchive.nationalarchives.gov.uk/+/www.dh.gov.uk/en/Healthcare/Longtermconditions/DH_064569.

^b^£15 was paid at the time of interview and £5 for the follow-up.

^c^We constructed two measures of the extent to which each patient’s network had changed across the 12-month period. The first was a binary measure (yes/no) indicating whether or not a network had lost one or more members considered important (indicated by being positioned in either the central or middle circle of the network) by the patient at time period 1. The second measure was the sum total across all network members of all the work done at time 1 by people no longer in the network at time 2. Both of these measures are indicative of loss of either people or work from the networks.

From the regression results for each type of work, we conducted a post-estimation omnibus test to determine if amounts of work undertaken differed significantly across relationship categories. Where it did we compared the mean score for weak ties against the mean score for each of the other types of ties. All analysis was conducted using STATA (version 11 [31]) and an alpha-level of 5%.

^d^A limitation of the postal-questionnaire follow up method was that that the reporting of the accumulation of new ties was likely to be an underestimate.

Strong ties (those placed within the inner circle) had the highest mean ‘work’ scores in the emotional, practical and illness types of work. In all but ‘practical’ work weak ties scored significantly lower than stronger ties. In this type of work, there was no statistical difference between weak ties and those placed in the middle circle of importance.

^e^Relational work has been used to describe the tasks which are required to develop and sustain interpersonal relationships and is seen as easily influenced to movements and dynamics within workgroup subsystems [32].

## Competing interests

Anne Rogers is Associate Editor of Implementation Science. All other authors declare they have no competing interests.

## Authors’ contributions

AR, IV, CB, DR designed the study; HB, IV, AK, CB collected data. All authors were involved in the analysis of data. DR and HB undertook the quantitative social network analysis, and AR, IV, CB, IV and HB undertook the qualitative analysis. AR drafted the paper, and HB, IV, AK and CB iterated and commented on drafts. All authors read and finalised the manuscript.
